# Ethical Issues Associated With Routine Screening and Prophylaxis for Group B Streptococcus in Pregnancy

**DOI:** 10.1155/S1064744996000099

**Published:** 1996

**Authors:** Michael K. Yancey, Anne Schuchat, Patrick Duff

**Affiliations:** 1 Department of Obstetrics and Gynecology MCHKOB 1 Jarrett White Road Tripler Army Medical Center HI 96859-5000 USA; 2 Department of Obstetrics and Gynecology University of Florida College of Medicine Gainesville FL USA; 3 National Center for Infectious Diseases Centers for Disease Control and Prevention Atlanta GA USA

## Abstract

An increased awareness of the impact of group B streptococcus (GBS) infection on neonatal outcome has prompted several seemingly discordant committee recommendations. Intrapartum antibiotics are effective in reducing the risk of neonatal morbidity when administered to a colonized woman who has a clinical condition that places her neonate at high risk for early-onset sepsis. However, less is known about the efficacy of prophylactic antibiotics in the colonized woman who does not have obvious risk factors. Some authorities have suggested that providers refrain from administering intrapartum antibiotics to colonized women who do not have any of these risk factors, primarily due to concerns about potential adverse reactions, selection of resistant pathogens, and cost-effectiveness. These recommendations may conflict with the desires of an informed woman who weighs the real, albeit low, risk for serious neonatal disease against the lower perceived risk of adverse maternal sequelae from allergic reactions to the antimicrobial agents. Selective prophylaxis for GBS disease that is limited to the colonized parturient with risk factors has the potential for creating conflict because maternal beneficence-based obligations of the physician may be at odds with maternal autonomy-based obligations. We believe that, given all currently available information, providers have a moral obligation to discuss GBS screening and treatment issues with patients. The potential for conflict between patient and physician at the time of delivery can be minimized through the use of preventive ethics, allowing patients to develop advance directives regarding intrapartum management within the confines of reasonable and cost-effective care. Until a consensus is reached among experts, the most prudent approach would be to address such issues proactively and individualize care based upon the overall estimation and anticipation of risk as well as the patient's specific desires.


Infectious Diseases in Obstetrics and Gynecology 4:36–42 (1996)
© 1996 Wiley-Liss, Inc.

### Ethical Issues Associated With Routine Screening and Prophylaxis for Group B Streptococcus in Pregnancy

**Michael K. Yancey, Anne Schuchat, and Patrick Duff**

*Department of Obstetrics and Gynecology, Tripler Army Medical Center, HI (M.K.Y.); Department of Obstetrics and Gynecology, University of Florida College of Medicine, Gainesville, FL (P.D.); National Center for Infectious Diseases, Centers for Disease Control and Prevention, Atlanta, GA (A.S.)*

### Abstract

An increased awareness of the impact of group B streptococcus (GBS) infection on neonatal outcome has prompted several seemingly discordant committee recommendations. Intrapartum antibiotics are effective in reducing the risk of neonatal morbidity when administered to a colonized woman who has a clinical condition that places her neonate at high risk for early-onset sepsis. However, less is known about the efficacy of prophylactic antibiotics in the colonized woman who does not have obvious risk factors. Some authorities have suggested that providers refrain from administering intrapartum antibiotics to colonized women who do not have any of these risk factors, primarily due to concerns about potential adverse reactions, selection of resistant pathogens, and cost-effectiveness. These recommendations may conflict with the desires of an informed woman who weighs the real, albeit low, risk for serious neonatal disease against the lower perceived risk of adverse maternal sequelae from allergic reactions to the antimicrobial agents. Selective prophylaxis for GBS disease that is limited to the colonized parturient with risk factors has the potential for creating conflict because maternal beneficence-based obligations of the physician may be at odds with maternal autonomy-based obligations. We believe that, given all currently available information, providers have a moral obligation to discuss GBS screening and treatment issues with patients. The potential for conflict between patient and physician at the time of delivery can be minimized through the use of preventive ethics, allowing patients to develop advance directives regarding intrapartum management within the confines of reasonable and cost-effective care. Until a consensus is reached among experts, the most prudent approach would be to address such issues proactively and individualize care based upon the overall estimation and anticipation of risk as well as the patient's specific desires. © 1996 Wiley-Liss, Inc.

Key words
GBS screening, perinatal ethics, neonatal sepsis

Early-onset neonatal group B streptococcus (GBS) sepsis, which continues to be a major cause of neonatal morbidity and mortality, has recently emerged as a national public-health concern. Several professional organizations have published specific recommendations regarding the identification and subsequent treatment of GBS genital colonization in expectant women. In 1992, the American Academy of Pediatrics[1] recommended universal screening of all pregnant women with rectovaginal cultures at 26–28 weeks gestation and intrapartum chemoprophylaxis of women with conditions that place their neonates at "high risk" for early-onset sepsis such as preterm delivery, prolonged rupture of the membranes, intrapartum fever, or a previous child afflicted with early-onset disease. In contrast, the American College of

Address correspondence/reprint requests to Dr. Michael K. Yancey, Department of Obstetrics and Gynecology, MCHK-OB, 1 Jarrett White Road, Tripler AMC, HI 96859-5000.

**The opinions and assertions contained herein are the expressed views of the authors and are not to be construed as official or reflecting the opinions of the Department of Defense, the Department of the Army, or the Centers for Disease Control and Prevention.**

Received February 13, 1996
Accepted May 6, 1996

**Obstetricians and Gynecologists (ACOG)[2] recommended empiric treatment of parturients with risk factors, even though their colonization status was unknown. The Centers for Disease Control and Prevention (CDC)[3] recommends that providers adopt a strategy for the prevention of early-onset GBS sepsis and inform patients regarding the prevention strategy. One of two strategies is proposed. The first recommends intrapartum antibiotic prophylaxis for women who deliver prematurely (<37 week gestation) and offers intrapartum antibiotics to women identified as carriers through the collection of prenatal screening cultures at 35–37 weeks gestation, while the second strategy proposed is similar to the original ACOG recommendation cited above. Recently, public sentiment has driven several state legislators to propose legal mandates for universal management guidelines.**

Concern about the potential complications and cost associated with widespread use of intrapartum antibiotics has been cited as a reason to avoid the routine use of antimicrobials in women who are colonized with GBS but who lack identifiable risk factors. The absence of an endorsement by the ACOG or the American Academy of Pediatrics for the use of prophylactic antibiotics in this population was primarily due to the lack of relevant clinical information about the potential benefit of such agents in this population. However, since this group comprises the majority of colonized women, the provider must consider the implications of identifying such individuals through routine screening prior to adopting such a practice. The ACOG has stated that, if a screening test is performed, a woman should be informed of the test result and its potential implications in regard to use of intrapartum antibiotics for conditions that would place her newborn at high risk. During such a discussion, many parturients will intuitively inquire about the potential risk posed by her colonization status if none of the aforementioned high-risk conditions are present. Although the informed patient may recognize that her fetus is "low" risk, she is likely to consider "any" risk unacceptable and unlikely to be dissuaded by concerns about "public health" and "cost-effectiveness." When faced with the potential for serious harm to their newborns, most women will choose any reasonable measure to protect their infants from such harm, even at the risk of incurring personal risk or discomfort in the process. In the colonized parturient without risk factors, the primary issue will center on the potential benefits and risks of intrapartum prophylactic antibiotics. The role of maternal participation in the decision to administer intrapartum prophylaxis was not a focus of early GBS recommendations.

We will use the framework of ethical principles applied to perinatal issues, as described by Chervenak and McCullough,[4] to explore these issues. These authors have emphasized the concept of beneficience, suggesting that providers use all available clinical information after carefully analyzing the individual circumstances and issues facing the patient and select a management plan expected to result in the greatest balance of good over harm. Patient autonomy implies that each individual patient has unique values, experiences, and beliefs that enable her to arrive competently at a self-determining decision about her care. In attempting to determine if providers are justified in withholding therapy against the patient's desires, we will examine closely the potential good and potential harm associated with the use, or avoidance, of intrapartum prophylactic antibiotics in the colonized parturient without risk factors (Table 1). Finally, we will propose and describe an antenatal informed consent process that, hopefully, will enable patients and providers to develop a management plan that serves the best interests of all parties and prevents patient-provider discord.

### RISKS ASSOCIATED WITH INTRAPARTUM CHEMOPROPHYLAXIS

One of the principal concerns about the liberal use of intrapartum antibiotics is the potential for an increased incidence of serious perinatal infections due to resistant organisms such as *Escherichia coli*. Investigations in the routine administration of penicillin to newborn infants have failed to conclusively demonstrate an associated increase in the incidence of infections caused by penicillin-resistant organisms.[5,6] Similarly, studies examining the effects of intrapartum antibiotic use for prophylaxis of neonatal GBS disease have not demonstrated a significant increase in neonatal infections from nonstreptococcal organisms resistant to penicillin or ampicillin.[6–9] However, investigations examining this issue have reported results for a relatively small number of neonates, and large-scale studies of the overall impact of widespread intrapartum antibiotic use on perinatal infections due to resistant pathogens are

TABLE 1. Summary of potential risks and benefits associated with intrapartum GBS prophylaxis

| Risks or benefits | Rate | Reference |
|---|---|---|
| Risk of early-onset GBS disease in neonates born to women with prenatal GBS colonization and no intrapartum risk factors (in absence of intrapartum antibiotic prophylaxis) | ≈1 in 200 (5.1/1,000) | 18 |
| Risk of early-onset GBS disease in neonates born to women with negative prenatal GBS cultures and no intrapartum risk factors (in absence of intrapartum antibiotic prophylaxis) | ≈1 in 3,200 (0.3/1,000) | 18 |
| Risk of early-onset GBS disease in neonates born to women with prenatal GBS colonization and one or more intrapartum risk factors (in absence of intrapartum antibiotic prophylaxis) | ≈1 in 25 (41/1,000) | 18 |
| Efficacy of intrapartum antimicrobial prophylaxis against early-onset GBS disease | 97% | 20 |
| Reduction in total GBS-related health-care costs with one of the currently recommended prevention strategies | 25–64% | 12, 19 |
| Risk of allergic reactions to penicillin | 0.7–10% | 13 |
| Risk of anaphylaxis to penicillin | 0.015–0.004% (4–15/100,00) | 13 |
| Risk of fatality from anaphylactic shock among patients treated with penicillin (note: one-third of fatalities in persons who had previously reacted to penicillin) | 0.0015–0.002% (1–2/100,000) | 13 |
| Risk of increased antimicrobial resistance among bacteria causing perinatal infections following intrapartum antimicrobials | Not known; theoretic concern | |

currently not available. While widespread intrapartum antibiotics remain a legitimate theoretical concern, there is no current evidence that the liberal use of limited-spectrum penicillins in the peripartum period has adversely effected the spectrum of these agents or promoted the emergence of resistant organisms over the past several decades.[10]

The use of intrapartum antimicrobial agents may have a direct impact upon neonatal management, as some pediatricians may elect to begin empiric antimicrobial therapy or pursue additional laboratory evaluation of an asymptomatic neonate solely because of intrapartum antibiotic exposure.[11] The guidelines of the American Academy of Pediatrics[1] provide several recommendations for the management of asymptomatic newborns who have been exposed to intrapartum antibiotics. However, these recommendations, which are not based upon published trials, may not be acceptable to some pediatricians. Extensive neonatal laboratory evaluations or routine antimicrobial therapy for all infants exposed to intrapartum antibiotics would undoubtedly prolong hospitalization, having a negative impact on cost-effectiveness and the potential for good over harm. Therefore, the management practices of the individual neonatal providers need to be considered carefully prior to the administration of intrapartum antibiotics to colonized women without risk factors.

Some authors have estimated that a significant number of major adverse reactions, principally fatal analphylaxis, would occur annually if universal antibiotic prophylaxis were utilized for all GBS carriers.[12] The estimated risk of fatal anaphylaxis from penicillin-type agents is approximately 1:100,000; however, this estimate may not be directly applicable to a relatively young and healthy population of parturients who would be receiving these agents in an inpatient setting.[13] Nevertheless, there have been anecdotal reports of adverse reactions to antimicrobial agents prescribed for GBS prophylaxis that have resulted in serious morbidity.[14,15]

### RISKS ASSOCIATED WITH AVOIDANCE OF INTRAPARTUM CHEMOPROPHYLAXIS

The incidence of early-onset GBS sepsis in a neonate delivered to a colonized parturient without established risk factors is approximately 0.5%, and the mortality rate in the infected infant is about 2–3%.[7,12,16] In a recent population-based, case-control study of infants with early-onset GBS sepsis, Schuchat et al.[17] found that 77% of the cases were in term neonates, with approximately 25% of the bacteremic neonates having no established risk factors. In a large multicenter study of neonatal sepsis, Weisman et al.[16] similarly found a substantial percentage of cases of early-onset GBS sepsis in term neonates without risk factors and determined that such an infection increased the neonatal mortality rate 40-fold relative to term neonates without sepsis. However, while the majority of infections occur in term neonates, the overall attack rate in term infants without risk factors is relatively low compared with preterm neonates or term infants delivered to women with risk factors. With this low attack rate, the efficacy of intrapartum antibiotics in interrupting vertical transmission to these neonates remains unclear because appropriate randomized clinical trials with sufficient power to detect a benefit have not been done. Despite the lack of objective evidence, several investigators have suggested that the efficacy of such therapy is equivalent to, or superior to, treatment of the neonates with risk factors[7,8,18] and have proposed that such therapy be offered to all colonized parturients.[7,8]

### BENEFITS OF WITHHOLDING INTRAPARTUM CHEMOPROPHYLAXIS

The avoidance of intrapartum antibiotic therapy probably increases the likelihood that neonates delivered to colonized, "low-risk" parturients will have routine neonatal courses free of the encumbrance of laboratory evaluations and antibiotic therapy which might occur if their mother received intrapartum antibiotics.[8,11,14] The weight of this factor may vary considerably among institutions and neonatal providers dependent upon local management practices. Additionally, the avoidance of intrapartum antibiotics will reduce the possibility of adverse maternal reactions to medications and lower overall maternal hospital costs. However, 2 recent decision analyses have suggested that the treatment of all colonized parturients is still a cost-effective approach when the net costs of maternal and neonatal care of such a preventive strategy are applied to a large population.[12,19]

### BENEFITS OF ADMINISTERING INTRAPARTUM CHEMOPROPHYLAXIS

Current evidence supports the concept that the risk of acquiring neonatal and maternal infection from GBS is a continuum related to multiple factors, rather than an absolute categorical designation. Additionally, there is no biologic reason to expect that antibiotics are less efficacious in colonized women without risk factors than in women with prolonged rupture of the membranes, amnionitis, or preterm delivery, the bacterial inoculum present in the upper genital tract is likely to be substantially less in women without risk factors compared with those with amnionitis or prolonged rupture of the membranes. Therefore, the potential for successful prophylaxis may be greater in the former group. A recent meta-analysis concluded that there was a 30-fold reduction in the incidence of early-onset GBS neonatal infection associated with intrapartum antibiotic prophylaxis and that this risk reduction did not differ between subgroups of women with or without risk factors.[20] In contrast, a different group of investigators, using more stringent guidelines for critically reviewing published randomized, controlled trials, concluded that there is a lack of conclusive evidence that intrapartum chemoprophylaxis reduces perinatal GBS infections in any population.[9] Therefore, although the majority of studies suggest that the administration of intrapartum antibiotics reduces vertical transmission of GBS, the magnitude of the overall benefit for specific populations of parturients has not been defined.

If prophylaxis is routinely delayed until established risk factors are present, patients with protracted labor may develop intraamniotic infection, thus placing themselves and their infants at greater risk of morbidity. Yancey et al.[21] recently showed that lower-genital-tract GBS colonization was an independent risk factor for the development of chorioamnionitis. In turn, symptomatic intraamniotic infection subsequently increased the likelihood that the neonate would become infected in spite of intrapartum antimicrobial therapy. Moreover, Ascher et al.[22] recently reported a series of 96 neonates with GBS sepsis. Eighteen of the mothers had received intrapartum chemoprophylaxis and 16 of their neonates had early-onset disease, while the remaining 2 had late-onset infections. Of the 16 bacteremic neonates with early-onset disease after intrapartum antibiotic exposure, 13 were delivered to women who had been treated for chorioamnionitis. Both of these studies indicate that delaying antimicrobial therapy until the mother has a clinically apparent intraamniotic infection may reduce the effectiveness of therapy. Accordingly, one can postulate that antibiotic therapy early in the intrapartum period may reduce the incidence of ascending infection in colonized parturients, with a subsequent decrease in the incidence of early-onset neonatal sepsis and maternal chorioamnionitis; however, this premise remains unproven.

An additional benefit, which may be difficult to quantitate, is the avoidance of added apprehension for the parturient who will likely be aware of her colonization status and its potential implications. Our anecdotal experience suggests that this is a very real concern for parturients. While the overall benefit of intrapartum chemoprophylaxis remains debatable in a population of colonized women without risk factors, the majority of such women receiving prophylaxis find solace in the concept that they are pursuing an aggressive, rational means of preventing neonatal morbidity.

Finally, the liberal use of intrapartum antibiotics is likely to ease providers' concerns about potential legal recourse in the event of serious neonatal morbidity or mortality related to early-onset sepsis. While this factor should not be used as justification for the routine use of intrapartum antibiotics, recent attention directed at perinatal GBS infections by the legal community has undoubtedly had an impact on the prescribing practices of some providers.

### DISCUSSION

For this article, we chose not to examine the issue of whether a practitioner should obtain antenatal GBS screening cultures. Rather, we have focused on the potential for patient-provider conflict if a screening test is obtained and the colonized parturient does not have a clinical condition that would warrant classification as high-risk and prompt prophylactic antimicrobial therapy. In most obstetric populations, only 15–25% of the colonized women will have risk factors that would warrant intrapartum therapy under most currently published committee recommendations. Consequently, most colonized parturients, who will not develop intrapartum risk factors, are at risk for conflict with their providers unless there is a clear agreement about the specific intrapartum management plan. Controversy about the efficacy of prophylaxis, variations in patient demographics, and individual management practices currently preclude the development and application of a single management algorithm for the colonized parturient without risk factors. Clinically applied preventive ethics can provide a useful framework for meaningful discussions between patient and provider which should result in an understanding between both parties of the patient's preferences and the clinician's judgment in regard to intrapartum antimicrobial prophylaxis.

Physicians may elect to forego prophylactic therapy for low-risk women, motivated by beneficence-based issues related to the potential for adverse sequelae in their specific patients as well as the collective good of the population at large (reducing the potential for the emergence of resistant microbes and containing health-care costs). Viewed from a pure public-health perspective, withholding intrapartum therapy from "low-risk" women may seem appropriate. However, such a beneficence-based decision must be substantiated sufficiently by current clinical information to warrant overriding the patient's autonomy-based decision to receive intrapartum chemoprophylaxis. Conversely, providers should not intrusively assume that all colonized parturients without risk factors will desire intrapartum prophylaxis, as some women will likely view the potential for adverse maternal reactions or altered neonatal management as sufficient basis for declining therapy.

We do not feel that health-care cost containment should be used as a rationale for avoiding screening cultures or the subsequent withholding of antibiotics from colonized parturients. Investigations employing decision-analysis techniques have shown that prenatal screening and intrapartum prophylaxis of all colonized women is cost-effective when total health-care costs are considered.[12,19] The empiric intrapartum prophylaxis of unscreened parturients who are preterm or have prolonged rupture of the membranes, as suggested by the ACOG, has similarly been shown to be cost-effective by these investigations. Therefore, we suggest that GBS prevention be considered an integral and necessary part of prenatal care based upon the current evidence that such prevention is medically, morally, and fiscally justified.

A proposal for the application of preventive ethics has been previously applied to the issue of routine ultrasound in pregnancy with the systematic application of the informed consent process suggested as the primary means of resolution.[23] The suggestion that such an approach be adapted in the management of GBS colonization may seem intuitive to some; however, based upon recent practice surveys, substantial variation in management for parturients and neonates with respect to GBS screening and treatment is apparent.[24,25] Current management practices range from the rigid adherence to published guidelines, i.e., withholding intrapartum antimicrobial agents to colonized parturients without risk factors, to the paternalistic administration of intrapartum antibiotics to all colonized women without the allowance for maternal input in the decision to treat. This variation is likely due to several factors, some of which have global application such as the lack of consensus among experts regarding the ideal management approach and others which are locally important such as population demographics or neonatal management practices. Additionally, there are ever-looming concerns about the potential legal repercussions of either failing to provide intrapartum prophylaxis to all colonized women, particularly in light of the widely held premise that the majority of early-onset GBS infections are preventable, or a serious adverse reaction following the administration of antibiotics to a colonized woman without risk factors.

Each patient must be well informed in order to make an appropriate and reasonable decision regarding her care; thus, an ongoing dialogue between provider and patient is the best approach to preventing conflict.[4] The informed-consent process is a critical step in the course of empowering patients to make autonomous decisions. This process requires an unbiased presentation of factual information relevant to the circumstances and issues in a manner which optimizes the patient's understanding of the crucial points, followed by a proposal or recommendation by the provider of a management plan judged to provide the greatest potential good over harm and, finally, the subsequent acceptance or refusal of the plan by the patient. A formal, candid discussion and informed consent proceeding have the potential benefit of fostering patient-provider rapport by actively demonstrating respect for patient autonomy. A crucial step in the informed-consent process is to verify that the patient has a reasonable understanding of the relevant issues and an appropriate amount of time to arrive at an appropriate decision. Accordingly, the appropriate time for such a discussion is in the antenatal period, well before the anticipated onset of labor. Most importantly, addressing such issues antenatally will help avoid acute intrapartum conflicts between patient and provider. Additionally, the well-informed patient may be a safeguard against failure of a treatment protocol since she may serve as a valuable resource for prenatal culture results and previously discussed management plans in the event that prenatal records or data are unavailable at the time of admission.

We are not suggesting that providers acquiesce to unreasonable or unfounded consumer pressure. Rather, we suggest that, in the face of a paucity of decisive clinical data, clinicians should support and respect the parturient's ability to direct her treatment within acceptable clinical bounds. Importantly, beneficence-based concerns cannot be completely abandoned in favor of autonomy-based obligations, and patient care must remain within the confines of reasonable and cost-effective management. In the event that the provider disagrees with this course of action, he or she is certainly free to recommend and document a dissenting opinion. However, the failure to disclose all reasonable therapeutic alternatives impairs the women's ability to arrive at an autonomous decision suited to her own beliefs and perceptions. Therefore, the management issues surrounding GBS colonization cannot be ignored or trivialized. We suggest that providers take a proactive approach to this issue in caring for their patients, rather than passively waiting for patients to broach the subject. Additionally, we believe that women should be empowered with the necessary knowledge and opportunity to choose a reasonable management plan. Only through thoughtful and accurate discussions related to the issues surrounding GBS colonization can women and their physicians avoid unsettling conflict.

### REFERENCES

1. American Academy of Pediatrics: Guidelines for prevention of group B streptococcal infection by chemoprophylaxis. Pediatrics 90:775–778, 1992.
2. American College of Obstetricians and Gynecologists: Group B streptococcal infections in pregnancy. ACOG Technical Bulletin No. 170, July 1992; and Hankins GV, Chalas E: Group B streptococcal infections in pregnancy: ACOG's recommendations. ACOG Newsletter, May 1993.
3. Schuchat A, Whitney C, Zangwill K: Prevention of perinatal group B streptococcal disease: A public health perspective. MMWR 45:1–24, 1996.
4. Chervenak FA, McCullough LB: Clinical guides to preventing ethical conflicts between women and their physicians. Am J Obstet Gynecol 162:303–307, 1990.
5. Siegel JD, McCracken GHJ, Threlkeld N, Milvenan B, Rosenfeld CR: Single-dose penicillin prophylaxis against neonatal group B streptococcal infections: A controlled trial in 18,738 newborn infants. N Engl J Med 303:769–775, 1980.
6. Pyati SP, Pildes RS, Jacobs NM, Ramamurty RS, Yeh TF, et al.: Penicillin in infants weighing two kilograms or less with early-onset group B streptococcal disease. N Engl J Med 308:1383–1389, 1983.

7. Katz VL, Moos MK, Cefalo RC, Thorp JM, Bowes WA, Wells SD: Group B streptococci: Results of a protocol of antepartum screening and intrapartum treatment. Am J Obstet Gynecol 170:521–526, 1994.
8. Garland SM, Fliegner JR: Group B streptococcus and neonatal infections: The case for intrapartum chemoprophylaxis. Aust NZ J Obstet Gynaecol 31:119–122, 1991.
9. Ohlsson A, Myhr TL: Intrapartum chemoprophylaxis of perinatal group B streptococcal infections: A critical review of randomized controlled trials. Am J Obstet Gynecol 170:910–917, 1994.
10. Monif GRG (ed): Infectious Diseases in Obstetrics and Gynecology. Omaha: IDI Publications, 1993.
11. Mercer BM, Ramsey RD, Sibai BM: Prenatal screening for group B streptococcus. II. Impact of antepartum screening and prophylaxis on neonatal care. Am J Obstet Gynecol 173:842–846, 1995.
12. Rouse DJ, Goldenberg RL, Cliver SP, Cutter GR, Mennemeyer ST, Fargason CA: Strategies for the prevention of early-onset group B streptococcal sepsis: A decision analysis. Obstet Gynecol 83:483–494, 1994.
13. Idsoe O, Guthe T, Wilcox RR, DeWeck AL: Nature and extent of penicillin side-reactions with particular reference to fatalities from anaphylactic shock. Bull WHO 38:159–188, 1968.
14. Pylipow M, Gaddis M, Kinney JS: Selective intrapartum prophylaxis for group B streptococcus colonization: Management and outcome of newborns. Pediatrics 93:631–635, 1994.
15. Heim K, Alge A, Marth C: Anaphylactic reaction to ampicillin and severe complication in the fetus. Lancet 337:859–860, 1991.
16. Weisman LE, Stoll BJ, Cruess DF, Hall RT, Merenstein GB, Hemming VG, Fischer GW: Early-onset group B streptococcal sepsis: A current assessment. J Pediatr 121:428–433, 1992.
17. Schuchat A, Deaver-Robinson K, Plikaytis BD, Zangwill KM, Mohle-Boetani J, Wenger JD: Multistate case-control study of maternal risk factors for neonatal group B streptococcal disease. Pediatr Infect Dis J 13:623–629, 1994.
18. Boyer KM, Gotoff SP: Strategies for chemoprophylaxis of GBS early-onset infections. Antibiot Chemother 35:267–280, 1985.
19. Yancey MK, Duff P: An analysis of the cost-effectiveness of selected protocols for the prevention of neonatal group B streptococcal infection. Obstet Gynecol 83:367–371, 1994.
20. Allen UD, Navas L, King SM: Effectiveness of intrapartum penicillin prophylaxis in preventing early-onset group B streptococcal infection: Results of a meta-analysis. Can Med Assoc J 149:1659–1665, 1993.
21. Yancey MK, Duff P, Clark P, Kurtzer T, Frentzen BH, Kubilis P: Peripartum infection asociated with vaginal group B streptococcal colonization. Obstet Gynecol 84:816–819, 1994.
22. Ascher DP, Becker JA, Yoder BA, Weisse M, Waecker NJ, Heroman WM, Davis C, Fajardo JE, Fischer GW: Failure of intrapartum antibiotics to prevent culture-proved neonatal group B streptococcal sepsis. J Perinatol 13:212–216, 1993.
23. Chervenak FA, McCullough LB, Chervenak JL: Prenatal informed consent for sonogram (PICS): An indication for obstetric ultrasound. Am J Obstet Gynecol 161:857–860, 1989.
24. Gigante J, Hickson GB, Entman SS, Oquist NL: Universal screening for group B streptococcus: Recommendations and obstetricians' practice decisions. Obstet Gynecol 85:440–443, 1995.
25. Mercer BM, Ramsey RD, Sibai BM: Prenatal screening for group B streptococcus. I. Impact of antepartum screening and antenatal prophylaxis and intrapartum care. Am J Obstet Gynecol 173:837–841, 1995.
